# Genomic epidemiology of *Clostridioides difficile* sequence type 35 reveals intraspecies and interspecies clonal transmission

**DOI:** 10.1080/22221751.2024.2408322

**Published:** 2024-09-20

**Authors:** Yun Luo, Yu Chen, Shan Lin, Hui Hu, Xiaojun Song, Qiao Bian, Weijia Fang, Huoyang Lv, Qin Wang, Jianmin Jiang, Yi-Wei Tang, Dazhi Jin

**Affiliations:** a School of Biotechnology and Biomolecular Sciences, University of New South Wales, Sydney, Australia; b School of Laboratory Medicine, Hangzhou Medical College, Zhejiang, People’s Republic of China; c Key Laboratory of Biomarkers and In Vitro Diagnosis Translation of Zhejiang province, Zhejiang, People’s Republic of China; d Laboratory Medicine Center, Department of Clinical Laboratory, Zhejiang Provincial People’s Hospital, Hangzhou Medical College, Zhejiang, People’s Republic of China; e Department of Public Health Emergency Response, Zhejiang Provincial Center for Disease Control and Prevention, Zhejiang, People’s Republic of China; f Department of Medical Oncology, The First Affiliated Hospital, Zhejiang University School of Medicine, Zhejiang, People’s Republic of China; g Department of Clinical Laboratory, Zhuji People’s Hospital of Zhejiang Province, Zhejiang, People’s Republic of China; h Cepheid, Danaher Diagnostic Platform, Shanghai, People’s Republic of China

**Keywords:** *Clostridioides difficile*, ST35, whole genome sequencing, genome epidemiology, clonal transmission, virulence, sporulation capacity

## Abstract

*Clostridioides difficile* sequence type (ST) 35 has been found in humans and animals worldwide. However, its genomic epidemiology and clonal transmission have not been explored in detail. In this study, 176 *C. difficile* ST35 isolates from six countries were sequenced. Genomic diversity, clonal transmission and epidemiological data were analyzed. Sporulation and virulence capacities were measured. Four ribotypes (RT) were identified including RT046 (97.2%), RT656 (1.1%), RT427 (0.6%), and RT AI-78 (1.1%). Phylogenetic analysis of 176 ST35 genomes, along with 50 publicly available genomes, revealed two distinctive lineages without time-, region-, or source-dependent distribution. However, the distribution of antimicrobial resistance genes differed significantly between the two lineages. Nosocomial and communal transmission occurred in humans with the isolates differed by ≤ two core-genome single-nucleotide polymorphism (cgSNPs) and clonal circulation was found in pigs with the isolates differed by ≤ four cgSNPs. Notably, interspecies clonal transmission was identified among three patients with community acquired *C. difficile* infection and pigs with epidemiological links, differed by ≤ nine cgSNPs. Toxin B (TcdB) concentrations were significantly higher in human isolates compared to pig isolates, and ST35 isolates exhibited stronger sporulation capacities than other STs. Our study provided new genomic insights and epidemiological evidence of *C. difficile* ST35 intraspecies and interspecies clonal transmission, which can also be facilitated by its strong sporulation capacity.

## Introduction

*Clostridioides difficile* is a Gram-positive, anaerobic, spore-forming bacillus that can cause antimicrobial-associated diarrhea worldwide, with various symptoms collectively referred to as *C. difficile* infection (CDI) [1]. CDI can be classified by origin as community-acquired CDI (CA-CDI) or hospital-acquired CDI (HA-CDI) [1]. In 2017, CDI accounted for approximately 223,900 infections and 13,000 deaths in the United States, with about 50% of cases being CA-CDI [2]. A recent multi-country CDI study reported that the prevalence of CA-CDI was 16.5% in the Asia-Pacific region [3]. Similarly, a study from one province in China found a relatively high prevalence of CA-CDI, at approximately 14.1% [4]. It is important to identify the reservoirs of CA-CDI from various sources and determine its clonal transmission across different hosts.

It is well known that humans and animals share the same genotypes of *C. difficile* worldwide, suggesting that animals may serve as a reservoir for *C. difficile*, with clonal transmission occurring reciprocally between animals and humans, potentially leading to CA-CDI [3,5–8]. Hypervirulent *C. difficile* genotypes, such as sequence types (ST) 1 and ST11, have been found in both humans and various animals, with potential interspecies clonal transmissions [9,10]. In recent years, *C. difficile* ST11 has emerged as one of the most significant pathogens associated with CA-CDI [10]. It has been reported that the molecular epidemiology of *C. difficile* differed between China and other countries in Asia, particularly in terms of predominant genotypes and antimicrobial resistance patterns [3,11,12]. ST35 or RT046 has been one of the dominant *C. difficile* genotypes in patients with CDI in China [13,14], especially in CA-CDI [4], and has been responsible for a minor nosocomial outbreak [15]. This genotype has been detected in patients with CDI, the farm environment, and pigs in both China and Sweden [16–19]. Possible clonal transmission between humans and pigs has been found in Sweden, although no general transmission pattern was identified [18]. Therefore, the genomic epidemiology and clonal transmission of this genotype should be further explored in detail.

Toxin A (TcdA) and B (TcdB) encoded by the *tcdA* and *tcdB* genes respectively are the main virulence factors in *C. difficile* [20]. TcdB is a cytotoxic toxin that is essential for virulence of *C. difficile* and mainly mediates intestinal inflammation [20]. As an obligate anaerobic pathogen, *C. difficile* can survive outside of hosts in the aerobic environment by forming dormant spores [21]. The pathogenesis of *C. difficile* also depends on the formation of aerotolerant dormant spores, allowing it to persistent within hosts and spread through human-to-human contact, human-to-animal contact, or environmental contamination [21]. Therefore, the spore form is considered the vehicle for CDI transmission. Furthermore, strong sporulation capacity facilitates CDI epidemics [21]. As reported, the hypervirulent ST1/RT027 produced significantly more toxins and presented a higher sporulation capacity than other genotypes, contributing to its global dissemination over the past two decades [20]. However, there is limited data on biological characteristics of other zoonosis-associated genotypes.

In this study, 176 *C. difficile* ST35 isolates obtained from humans and pigs across six countries were sequenced. Phylogenetic analysis, along with epidemiological data and phenotypic studies, was performed to provide evidence of the intra- and interspecies clonal transmission and to gain a better understanding of the sporulation and virulence capacities of *C. difficile* ST35 isolates.

## Methods

### Collection of isolates and epidemiological data

176 *C. difficile* ST35 isolates were collected from 2010 to 2021, including 13 isolates from pigs in six farms located in Hangzhou, Jiaxing, Shaoxing and Liaoyang in China. 163 isolates were collected from human. 13 human isolates were collected from South Korea, Singapore, Australia, USA and Japan in our previous study [11], 18 were collected from healthy infants in our unpublished study of *C. difficile* colonization in children, and the rest 132 were collected from patients with diarrhea in China (Table 1, Table S1). All isolates were recovered on cefoxitin-cycloserine fructose agar plates (Oxoid, Basingstoke, UK) at 37 ℃ for 48 h in an anaerobic chamber with GENbag Anaer (bioMérieux, Marcy l’Etoile, France). Epidemiological data of pigs and patients with CDI were collected and reviewed with the approval of the Ethics Committee of the Hangzhou Medical College (LL2022-01). Written informed consent was waived due to the retrospective nature of this study. A case patient who had CDI symptoms onset within 48 h after hospitalization or without hospitalization would be classified as CA-CDI, and the patient who presented CDI symptoms over 48 h after hospitalization would be classified as HA-CDI [1]. 50 publicly available *C. difficile* ST35 genomes with metadata were downloaded from the NCBI database (Table S1). Eighty ST2 and ST37 isolates from our previous study [14] were used for measuring TcdB concentration and sporulation capacity.

**Table 1.** Distribution of the 176 *C. difficile* ST35 isolates involved in this study.

Source	Isolates (%)
Host	
Diarrhea human	145 (82.4%)
Healthy infant	18 (10.2%)
Pig	13 (7.4%)
Isolation place	
China	
Zhejiang	93 (52.8%)
Hangzhou	55 (31.3%)
Jiaxing	8 (4.5%)
Jinhua	1 (0.6%)
Lishui	5 (2.8%)
Taizhou	2 (1.1%)
Wenzhou	2 (1.1%)
Ningbo	3 (1.7%)
Shaoxing	17 (9.7%)
Hebei	
Shijiazhuang	28 (15.9%)
Shanghai	20 (11.4%)
Beijing	8 (4.5%)
Hong Kong	5 (2.8%)
Liaoning	
Liaoyang	3 (1.7%)
Ningxia	
Yinchuan	3 (1.7%)
Shandong	
Zibo	1 (0.6%)
Sichuan	
Chengdu	1 (0.6%)
Guangdong	
Guangzhou	1 (0.6%)
South Korea	
Pusan	2 (1.1%)
Singapore	
Singapore	2 (1.1%)
Australia	
Sydney	2 (1.1%)
Perth	2 (1.1%)
USA	
New York	3 (1.7%)
Japan	
Fukuoka	2 (1.7%)

### PCR ribotyping

Three *C. difficile* isolates from our laboratory, including a hypervirulent RT027/ST1 and two animal-related genotypes (RT078/ST11 and RT AI-53/ST48), were compared in this study. Genomic DNA was extracted from the 176 isolates using the DNeasy Blood & Tissue Kit (Qiagen Inc., Valencia, CA, USA). Ribotyping was performed using PCR combined with capillary gel electrophoresis [22]. Data were submitted to the WEBRIBO database (https://webribo.ages.at/) for RT assignment. The ribotyping profiles were generated using the R package ggtree [23] and analyzed using the Unweighted Pair-group Method with Arithmetic Mean (UPGMA) [24].

### Whole genome sequencing (WGS)

The 176 *C. difficile* isolates were sequenced using the Hiseq X Ten platform with 150-base paired-end reads, and WGS libraries were prepared using TruePrep™ DNA library prep kit V2 (Illumina, San Diego, CA, USA) same as previously reported [7]. Adapters and low-quality sequences with default parameters, except for MINLEN set to 75, were removed using Trimmomatic v0.36 [25]. Raw reads were assembled using SPAdes v3.6.2 with the “careful” option [26]. In addition, one pig isolate (A9) was also sequenced by long reads sequencing using the GridION X5 (Oxford Nanopore Technologies, Oxford, UK). Canu v2.2 [27] and Unicycler v0.5.0 [28] were used to assemble and polish the complete genome A9. Analysis of *C. difficile* ST35 pan-genome was calculated using Roary v3.11.2 [29].

### Single-nucleotide polymorphism (SNP) calling and definition of genetic relationship among different isolates

SNPs were identified and called using a section of the SaRTree pipeline at a proportion threshold of 100 [30] against the complete genome A9 (RefSeq assembly accession: SRR18235872). Difference of core-genome SNPs (cgSNPs) was used to analyze genetic relationships among isolates according to the “gold standard” assay [31]. Each pair of isolates are considered to be a result of direct transmission if they differed by 0 to 2 cgSNPs and their isolation dates were separated by < 124 days, and each pair of isolates are genetically close if they differed by ≤ 10 cgSNPs [31].

### Phylogenetic analysis

A maximum likelihood (ML) tree was constructed using IQ-Tree [32] with 1,000 ultrafast bootstrap pseudoreplicates under default parameters (Best-fit model: TVM + F + ASC + R2) for phylogenetic analysis of 176 genomes sequenced in this study and 50 genomes downloaded from the NCBI. The ST3 strain DSM1296 (RefSeq assembly accession: GCF_000438845.1) was used to root the ML tree, which was curated using iTOL v6.6 (https://itol.embl.de/). Antimicrobial resistance (AMR) genes, virulence genes, and plasmid replicons were predicted using the ABRicate pipeline (https://github.com/tseemann/abricate) with NCBI AMRFinderPlus [33], Virulence Factors Database [34], and PlasmidFinder database [35], respectively, with the cutoff value > 80% for both gene coverage and identity as previously stated [19]. Sequences of *tcdB* gene were typed according to our previous study [36]. Sequences of *gyrA*, *gyrB*, and *rpoB* genes in *C. difficile* ST35 genomes were compared with that of *C. difficile* 630 genome (GenBank: CP010905.2), and their corresponding amino acid substitutions were analyzed.

### Measurement of *C. difficile* TcdB concentration and sporulation capacity

TcdB concentration was quantified as previously reported [37]. All 176 *C. difficile* isolates were cultured in brain heart infusion (BHI) broth at 37 ℃ for 48 h in an anaerobic chamber. TcdB concentration in BHI was measured and calculated using a formula according to a panel of purified TcdB standards (List Biological Laboratories, Campbell, CA, USA) with known concentrations ranging from 0.02 to 200 ng/μL.

Sporulation capacity was measured as previously reported [38]. Briefly, the 176 *C. difficile* isolates were cultured in BHI broth at 37 ℃ for 48 h in an anaerobic chamber. BHI cultures adjusted to approximately OD_600_ = 1 were heated at 60 ℃ for 25 min and cultured on BHI agar plates with 2% agar and 0.1% taurocholate. After 48 h of anaerobic culture, colony-forming units were counted.

All above experiments were repeated three times independently. As a limitation, the TcdA concentration was not quantified in this study due to the unavailability of TcdA standards.

### Statistics analysis

Data were analyzed using the Statistical Package for Social Sciences (v22.0; SPSS, Armonk, NY, USA). Results on TcdB concentrations and sporulation capacity were compared with data on different STs and locations using the parametric methods (analysis of variance and *t*-test). Results on TcdB concentrations and sporulation capacity were compared with data on different sources and lineages using non-parametric tests. Differences were considered statistically significant when *P* values were < 0.05.

### Data availability

Raw reads have been submitted to NCBI under BioProject accession numbers: PRJNA591265 and PRJNA811705. The complete genome A9 has been submitted to the NCBI SRA database (accession number: SRR18235872).

## Results

### Comparative analysis of PCR ribotyping profiles among *C. difficile* ST35 isolates

**Figure 1.**
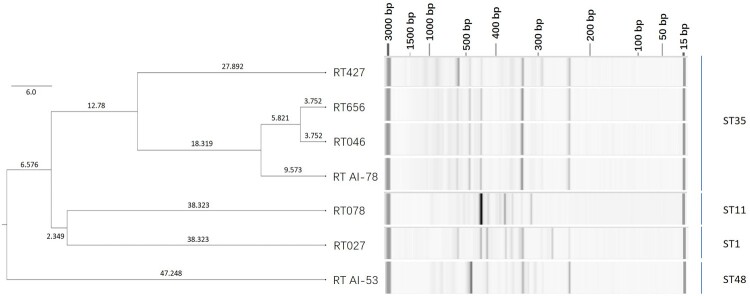
Dendrogram of seven ribotyping patterns by capillary gel electrophoresis. The dendrograms were clustered using UPGMA. The length of branch was labelled above each branch.

Ribotyping was performed for the 176 isolates. 171 (97.2%) isolates were identified as RT046, two (1.1%) as RT656, another two (1.1%) as RT AI-78, and one (0.6%) as RT427, showing that ST35 were not entirely consistent with RT046. The dendrogram of ribotyping profiles showed that RTs 046, 427, 656, and AI-78 were grouped into one cluster, which differed from RTs 027, 078, and AI-53 (Figure 1).

### Genome diversity and phylogenetic analysis of ST35 genomes

ST35 pan-genome comprised 141,685 genes at *n* = 226, including 2,808 core genes (99% ≤ genomes ≤ 100%), 424 soft core genes (95% ≤ genomes < 99%), 1,035 shell genes (15% ≤ genomes < 95%), and 137,418 cloud genes (0% ≤ genomes < 15%). The core-genome accounted for 1.98% of the total gene repertoire. The pan-genome increased in size unboundedly along with an increased number of involved genomes. Conversely, the core-genome curve showed stable, and the number of core genes remained constant regardless of the number of genomes added (Figure S1). No specific accessory genes were found from human and pig isolates.

**Figure 2.**
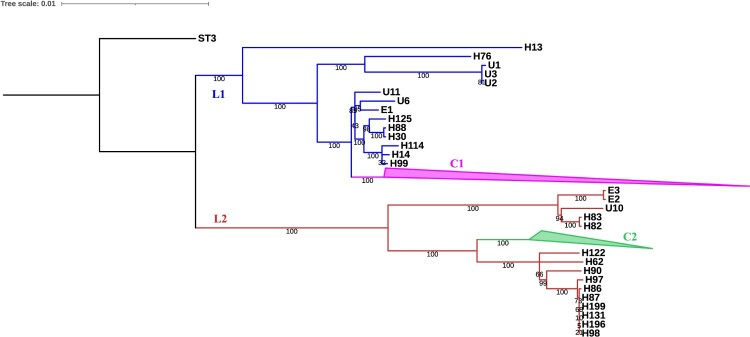
Maximum likelihood tree of 226 *C. difficile* ST35 genomes. The bootstraps were marked on each branch. Lineage 1 (L1) and lineage 2 (L2) were marked with blue and red full lines. Two main clusters (C1 and C2) were collapsed to reduce figure size.

Lineage 1 (L1) and Lineage 2 (L2) were identified on the ML tree. Two clusters (C1 and C2) were found in L1 and L2 respectively (Figure 2). A total of 60 genomes including the reference genome A9 belonged to C1, and 136 genomes were clustered into C2 (Figure S2). Two clone groups (CG1 and CG2) were found in C2. CG1 contained seven pig isolates and one human isolate from Hospital D. CG2 contained ten human isolates from Hospital A and one human isolate from Hospital D. No distinct time-, region-, or source-dependent distributions were observed in these two lineages by reviewing the epidemiological data.

1407 and 257 SNPs were located on genes and intergenic regions respectively. Excluding the 499 SNPs on hypothetical protein genes, a total of 908 SNPs were found on 653 genes. Of these genes, 477 genes had only one SNP, 128 genes harbored two SNPs, 32 genes had three SNPs, nine genes had four SNPs, three genes had seven SNPs, and two genes each had five and six SNPs (Table S2).

### Distribution of virulence genes, plasmid replicons and AMR elements in *C. difficile* ST35 genomes

Virulence genes and plasmid replicons were predicted in the 226 *C. difficile* ST35 genomes. All genomes harbored the *tcdA* and *tcdB* genes, and no binary toxin genes were found (Table S3). All the *tcdB* genes in this study were typed to *tcdB*1a. One SNP (C to T) at position 6,393 bp on the *tcdB* gene was found in all L1 isolates except H13 from Australia. No lineage-dependent sequence differences were found on *tcdA*, *tcdC*, *tcdE*, *tcdR*, and *cdd1* located in the pathogenicity locus (PaLoc).

A total of 57 types of plasmid replicons were identified in 215 *C. difficile* ST35 genomes, no plasmid replicons were found in the other 11 genomes (Table S4). *Rep*1_6_repE and *Rep*US43_1_CDS12738 were found in 87.6% (*n* = 198/226) and 93.8% (*n* = 212/226) of the ST35 genomes.

Analysis of AMR elements showed that all ST35 genomes possessed the vancomycin resistance gene (*vanG_Cd_*) as well as the intrinsic broad-spectrum class D *β*-lactamase-encoding gene (*bla*CDD-1). The tetracycline resistance gene *tet(M)* and macrolide-lincosamide-streptogramin B (MLS_B_) resistance gene *erm(B)* were found in 89.8% (*n* = 203/226) and 82.7% (*n* = 187/226) of the genomes, respectively. Two aminoglycoside resistance genes, aminoglycoside O-phosphotransferase (*aph (2”)-Ih*) and aminoglycoside 6-adenylyltransferase (*aadE*), were present in 88.9% (*n* = 201/226) and 26.5% (*n* = 60/226) of genomes, respectively. The chloramphenicol resistance gene (type A-11 chloramphenicol O-acetyltransferase, *catD*) was found in 26.1% (*n* = 59/226) of the genomes. *aadE* and *catD* were only found in C1 and C2. Mutations conferring fluoroquinolone resistance were present in the *gyrA* gene (amino acid substitution: T82A [0.9%, *n* = 2/226] and T82I [20.4%, *n* = 46/226]) and the *gyrB* gene (amino acid substitution: V426N, 77.0%, *n* = 174/226). The mutations gyrA_p. T82A and gyrA_p.T82I were only found in C1 and C2. Rifampin resistance associated mutations were present in the *rpoB* gene (amino acid substitutions: R505K [5.8%, *n* = 13/226] and I548M [4.0%, *n* = 9/226]), which were all found in C1. However, other genomes outside C1 and C2 carried few AMR genes, except *bla*CDD-1 and *vanG_Cd_* (Figure 2).

### Genomic epidemiology and clonal transmission of *C. difficile* ST35 genomes

**Figure 3.**
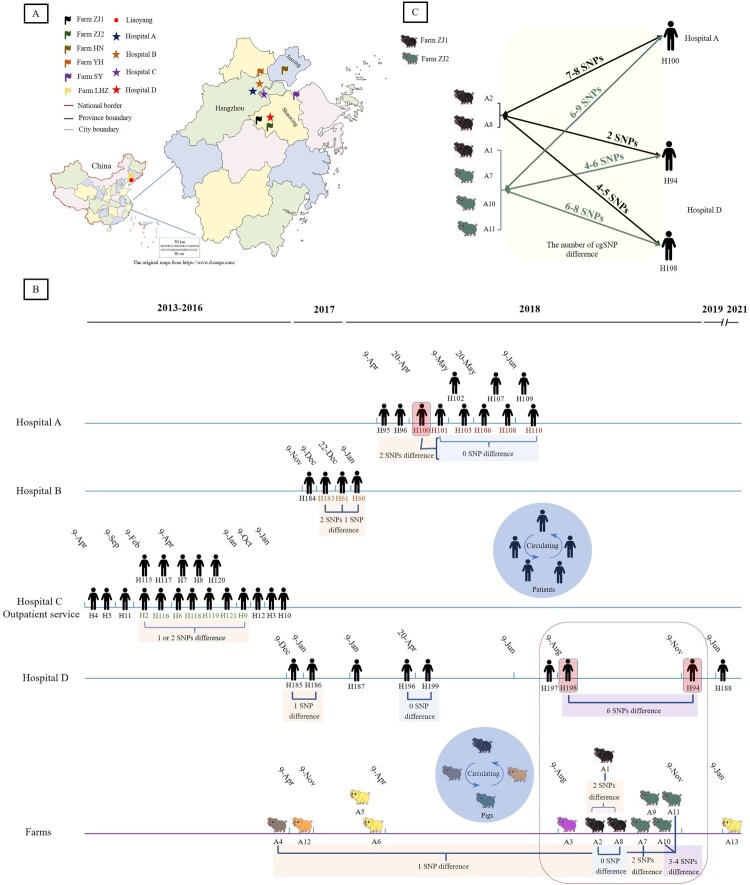
Genetic relationships among *C. difficile* ST35 isolates determined by cgSNP analysis. A: geographical locations of farms and hospitals; B: timelines of *C. difficile* ST35 isolates from four hospitals and different farms. Patients with CDI on the same blue line were admitted to the same hospital, pigs with different colors indicated different farms. The red numbers represented six patients from the same department. The light orange, purple, and blue frames illustrated the difference in cgSNPs between the isolates; C: the numbers of cgSNP difference were shown among nine isolates including three human and six pig isolates.

The number of SNP differences in pairs was determined for all 226 *C. difficile* ST35 genomes (Table S5). The genetic relationship of 55 *C. difficile* ST35 isolates from six farms and four hospitals respectively were analyzed (Figure 3A). Nosocomial and communal intraspecies clonal transmissions were found (Figure 3B). The 11 isolates obtained < 124 days apart in Hospital A had ≤ 10 cgSNP differences from each other. Among those differences, only one cgSNP differed between isolates H95 and H96. Six isolates, which carried the same AMR elements and the same replicons of the *Col* group, from the same floor in the Department of Oncology had ≤ two cgSNP differences. Among the four isolates from Hospital B, three had a difference of ≤ three cgSNPs with isolation time within < 124 days. All the isolates from Hospitals A and B except H100 were obtained from the patients classified as HA-CDI. Eighteen isolates were obtained from infant stool samples in the outpatient service in Hospital C from 2013 to 2016. The pairwise difference of one or two cgSNPs was found among seven of these isolates. Nine isolates were found in Hospital D, and their cgSNP differences varied from 0 to six SNPs. Notably, six isolates from Hospital A had ≤ 10 cg SNPs differed from 18 isolates from Hospital C. However, no direct epidemiological links were found among them.

Interspecies clonal transmission was observed between human and pig isolates (Figure 3C). All pig isolates in CG1, except A4, were collected from different farms within 124 days (Table S1). No cgSNP differences were found between A2 and A8, both of which had ≤ two cgSNP differences from A1, A4, and A7. We also found that human isolate H94 from Hospital D differed in two cgSNPs from both A2 and A8 while differed in four*-*six cgSNPs from other pig isolates except for A4 in CG1. In addition, human isolates H100 and H198 were genetically close to six pig isolates with differences in six-nine and four*-*eight cgSNPs, respectively.

### Comparison of TcdB concentration and sporulation capacity among different isolates

**Figure 4.**
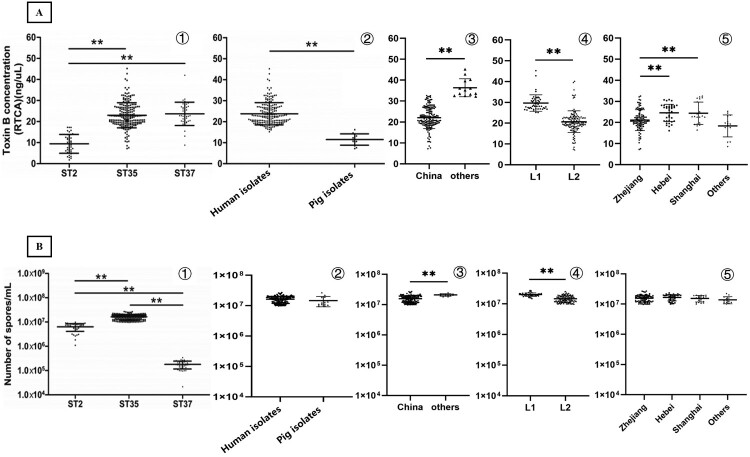
Comparison of TcdB production and determination of sporulation among the different groups. A: TcdB concentrations measured using the real-time cell analysis; B: sporulation capacity measured using the heat-induced method. **represented significant difference (*P *< 0.01). ZJ: Zhejiang, HB: Hebei, SH: Shanghai.

TcdB concentrations and sporulation capacities of the 176 ST35 isolates were measured in this study and compared to the 80 ST2 and ST37 isolates (Figure 4). The average concentration of TcdB secreted by ST35 isolates was 23.08 ± 6.35 ng/μL, which was significantly higher than that secreted by ST2 isolates (9.41 ± 4.48 ng/μL; *P *< 0.001), but was not statistically different from the TcdB produced by ST37 isolates (23.53 ± 6.29 ng/μL; *P *= 0.593). The TcdB concentrations were compared by different sources, locations, and lineages, respectively. The TcdB produced by human ST35 isolates was significantly higher (24.01 ± 5.59 ng/μL) than pig ST35 isolates (11.51 ± 2.71 ng/μL; *Z *= *–*5.98, *P *< 0.001). The TcdB concentration in ST35 isolates was significantly lower in China (22.04 ± 5.17 ng/μL) than that in the other countries (36.36 ± 4.27 ng/μL; *P* < 0.001). The TcdB concentration in L1 isolates (29.69 ± 3.96 ng/μL) was significantly higher than in L2 isolates (20.70 ± 5.24 ng/μL; *Z* = *–*8.94, *P* < 0.001) (Figure 4A).

The sporulation capacity of ST35 isolates (1.61 ± 0.41 × 10^7^ spores/mL) was significantly stronger than ST2 (6.33 ± 2.22 × 10^6^ spores/mL) and ST37 (1.81 ± 0.65 × 10^5^ spores/mL) (*F *= 386.06, *P *< 0.001) isolates. The ST35 isolates from China (1.57 ± 0.41 × 10^7^ spores/mL) had significantly lower sporulation capacity than those from other countries (2.09 ± 0.19 × 10^7^ spores/mL) (*t *= *–*8.45, *P* < 0.001). The sporulation capacity of the L1 isolates (2.01 ± 0.26 × 10^7^ spores/mL) was significantly stronger than those of the L2 isolates (1.47 ± 0.37 × 10^7^ spores/mL) (*Z* = *–*7.72, *P* < 0.001). No significant difference in the sporulation capacity was found between human and pig ST35 isolates (*t *= *–*1.39, *P *= 0.17) (Figure 4B).

## Discussion

Genomic evolution, clonal transmission, and biological characteristics have been well described for several *C. difficile* genotypes [39]. *C. difficile* genotypes shared in different hosts may serve as potential agents for reciprocal zoonotic transmission [8,9]. Although *C. difficile* ST35 or RT046 has been found in both humans and animals in China and Sweden [16,18,19], and possible clonal interspecies transmission has been confirmed by WGS, no general pattern of zoonotic transmission between humans and pigs has been identified [18]. In this study, the genomic epidemiology and genetic diversity of ST35 were further discussed. Zoonotic links have been revealed by genomic analysis, combined with detailed epidemiological data of different hosts. However, a limitation of this study is the small sample size and limited geographical distribution. The inference of this study may be affected by an enlarged sample size from other parts of the world. Nevertheless, it is possible that ST35 is a potential global zoonotic lineage, similar to ST11 and RT014, which have been widely recognized as zoonotic lineages in previous studies worldwide [7,8,9,10].

Although *C. difficile* ST35 or RT046 was not a predominant genotype in a European multicenter prospective study of CDI in hospitalized patients with diarrhea [40], nosocomial CDIs caused by this genotype have been reported in Sweden and Poland [16,41]. In China, ST35 was also associated with nosocomial CDIs [15], identified as one of the predominant genotypes causing CDI in hospitalized patients from different wards in hospitals in Zhejiang [19,42–44], and shared by both patients and animals in the same region [19]. Therefore, four tertiary hospitals and five nearby farms in Zhejiang were selected for this study, along with one farm in Liaoyang, where piglets originated from the same five farms in Zhejiang. Our study revealed that *C. difficile* ST35 was not only responsible for direct human-to-human transmission in hospitals, and pig-to-pig transmission in farms, but also for interspecies clonal transmission in China. As shown in Figure 3B, we found that most of the isolates with epidemiological links in Hospitals A, B, and D were clustered into C2. Seven isolates from infants without direct exposure at the outpatient service in Hospital C differed in only one or two cgSNPs. The findings demonstrated the nosocomial and communal intraspecies clonal transmitting and circulating in China. Notably, there was a difference of ≤ nine cgSNPs between six pig isolates from Farms ZJ1 and ZJ2 in CG1 and six human isolates from the same department in Hospital A, where one of the patients (H100) worked at Farm ZJ1. In addition, two other patients from Hospital D (H94 and H198) frequently purchased raw pork originating from farms ZJ1 and ZJ2 and handled pork meat with their bare hands without washing afterward. These three patients were CA-CDI cases with diarrhea within 48 h after hospitalization. The isolation dates of H94, H198, and six pig isolates were separated by < 124 days, whereas the isolation dates of H100 and those six pig isolates were separated by > 124 days. However, all these isolates had epidemiological links and belonged to the same cluster (C2). It is possible that the pig isolates were initially transmitted to the farm worker (the patient H100) due to occupational exposure. CDI may have developed after one day of chemotherapy, and finally leading to a nosocomial CDI transmission in Hospital A. Moreover, the hand-to-mouth route was a possible way for the bacteria to be directly transmitted between pigs and humans (patients H94 and H198). Therefore, our findings suggested that interspecies clonal transmission occurred through either food production or workplace exposure.

Previously reports have shown that certain *C. difficile* STs, such as ST37, ST29, and ST81, corresponded well to specific RTs [14]. However, our study revealed a highly exclusive correlation between ST35 and RT046. Among the 176 isolates confirmed as ST35, RT046 was the predominant ribotype, however three other RTs (RT656, RT427, and RT AI-78) were also identified. This finding highlights the inconsistency between PCR ribotyping and multilocus sequence typing. It has also been reported that *C. difficile* associated with zoonosis, such as RT014 and ST11, exhibit highly diverse evolutionary genomes [7,10]. Therefore, further studies on ST35 isolates are needed to analyze the diversity and phylogeny of RTs.

Like other *C. difficile* genotypes [39], *C. difficile* ST35 genomes also showed high levels of genetic diversity and remarkable plasticity, containing various AMR and exogenous genes. The core-genome represented 1.98% of the pan-genome in ST35 genomes, which was lower than the ratios in *C. difficile* ST11 (19.8%) and RT014 (30.3%) [7,10]. The lower core-genome suggested that ST35 may experience more frequent gene transfer and homologous recombination events than in other genotypes. Many exogenous genes were associated with metabolism, adherence, biosynthesis, pathogenesis, biofilm formation, and signal transduction. These genes may contribute to maintaining the biological activity of the bacteria, allowing them to survive better in challenging suboptimal environments [39]. Since the *C. difficile* evolutionary rate is about 1.4 mutations per genome per year [39], the possibility of two SNPs occurring on the same gene is low. In this study, a total of 176 genes harbored more than one SNP. Furthermore, we found that the nonsingleton SNPs on 130 of these genes were not located on the same branches (Table S2, Figure S3). It suggested that these SNPs resulted from independent mutation events. We assumed that ST35 genomes experienced higher levels of genetic mutations which may make it to be a zoonosis-associated genotype.

There are limited publications on AMR elements in *C. difficile* ST35 genomes. Our study showed that the vancomycin resistance gene *vanG_Cd_* has been found in all genomes, however all isolates remained susceptible to vancomycin (data not shown), consistent with findings from other studies [7,10,38,45]. Additionally, all genomes in this study harbored the intrinsic *bla*CDD-1 gene, which mediates resistance to *β*-lactam antimicrobials, posing a serious potential threat [46]. The distribution of other AMR elements significantly differed between the two lineages on the ML tree. Two mutations in the *rpoB* gene conferring rifampin resistance were found exclusively in C1 isolates. Although rifampin is rarely associated with CDI, the use of its derivatives in CDI treatment has been linked to a CDI outbreak caused by a *C. difficile* RT046 clone in tuberculosis patients [41]. The emergence of ST35 isolates with mutations in the *rpoB* gene raises concern in public health, particularly during anti-tuberculosis chemotherapy.

It has been reported that *C. difficile* strains presented high-level resistance to fluoroquinolone, tetracycline, erythromycin, and other antimicrobials worldwide due to the relevant AMR elements [39]. A majority of ST35 genomes harbor three AMR genes including tetracycline resistance gene *tet(M)*, MLS_B_ resistance gene *erm(B)*, aminoglycoside resistance genes *aph (2”)-Ih*, along with the fluoroquinolone resistance mutation *gyrB*_p.V426N. These characteristics differ from those of *C. difficile* ST37 [38] and ST2 [45]. Tetracycline, erythromycin, streptomycin, and nalidixic acid were first used to treat bacterial infections in humans and animals around 70 years ago [47–50]. Since then, they have become some of the most widely used veterinary antimicrobials, particularly for promoting animal growth [51]. These four antimicrobials and their residues have been frequently detected in the environment [52–54]. Such frequent prescribing in human, common usage in animals, and wide distribution in the environment may provide continuously extensive selection pressure for *C. difficile* ST35 to acquire AMR elements for survival. The assumption was confirmed by a recent study that tetracycline selection pressure drives genome evolution in agriculture-associated *C. difficile* RT078 [49], suggesting that zoonosis-associated *C. difficile* genotypes may experience more adaptive genetic evolution due to more frequent gene homologous recombination, gene mutation, and horizontal gene transfer under varying circumstances.

TcdB plays an important role in the etiology of CDI [20], and its concentration is significantly correlated with the clinical severity of the disease [37]. All *tcdB* sequences in L1 differed from those in L2 by one single SNP at position 6,393 bp, while no lineage-dependent sequence differences were found in other genes in the PaLoc region. Our previous studies demonstrated that TcdB with different gene sequences exhibited varying toxic capacities [36], and the mutation on *tcdB* in *C. difficile* ST2 was associated with low TcdB expression [45]. Therefore, we hypothesize that the SNP (C to T) at position 6,393 bp may result in significantly higher TcdB production in L1 compared to L2 isolates. The TcdB production was found to be significantly higher in human isolates than in pig isolates, and isolates from China expressed less TcdB than those from other regions. Further studies are needed to uncover the molecular mechanisms driving the virulence shift in *C. difficile* ST35.

The dissemination capacity of *C. difficile* depends on the formation of aerotolerant spores, which allow for persistence within hosts, transmission through the human-to-human contact, environmental contamination, and facilitates efficient interspecies transmission [21]. In this study, we revealed that ST35 had a significantly stronger sporulation capacity than the other two main STs (ST2 and ST37) in China, indicating that ST35 isolates possess a high capacity of sporulation for adapting to aerobic environment and tolerating various harsh conditions. It may facilitate ST35 isolates to cause efficient interspecies clonal transmission from environments to humans or among different hosts through direct or indirect contact. However, this hypothesis requires further verification in ST35 and other zoonotic *C. difficile* genotypes.

## Conclusion

In conclusion, this study provided a detailed genomic analysis of *C. difficile* ST35 and described its evolution and genetic diversity. Furthermore, it revealed the intra- and interspecies clonal transmission of *C. difficile* ST35 in China. We also identified that the strong sporulation capacity of ST35 not only facilitated its survival in various environments but also contributed to the clonal intra- and interspecies transmission between humans and pigs. Our data extended the One Health concept of *C. difficile* by highlighting the connections between different hosts, offering deep insights into its molecular epidemiology, transmission, and genomic evolution.

## Supplementary Material

Supplementary Tables.xlsx

Fig.S1.eps

Fig.S2.eps

Fig.S3.eps

## Data Availability

Data of 50 ST35 genomes were available in the NCBI database with RefSeq assembly accession numbers in Table S1. Genomic data of the 176 isolates were deposited in NCBI under BioProject identifier numbers: PRJNA591265 and PRJNA811705. Data on epidemiological information were provided in Table S1.
